# Pathological fracture on osteolytic lesion

**DOI:** 10.11604/pamj.2021.39.280.25695

**Published:** 2021-08-30

**Authors:** Ousmane Traore, Mhamed Elyagoubi

**Affiliations:** 1 Département de Radiologie, Faculté de Médecine et d’Odonto-Stomatologie (FMOS), Bamako, Mali,; 2 Pediatric Radiology Department, Abderrahim EL Harouchi - Casablanca Children's Hospital

**Keywords:** Pathological fracture, X-ray, computed tomography

## Image in medicine

We report the case of a male child, aged three years, with no particular pathological history, who has presented for three months with isolated evasive lameness complicated two weeks before his admission by a total functional impotence of the right lower limb following a fall from his height. The clinical examination on admission found a child in good general condition, afebrile, with functional impairment of the right lower limb, associated with painful limitation of ipsilateral hip flexion. The blood count and C-reactive protein are normal. A frontal and lateral view of the right femur was performed (A, B) which revealed a proximal metaphyseal-diaphyseal lesion of the right femur, osteolytic (Ladwic type 2 geographic osteolysis), fairly well limited oval, containing thin partitions delimiting multiple cubicles. This gap blows the cortex which is partially ruptured on the external face of the bone with detachment of a thin cortical lamella intra-lesional testifying to a secondary fracture (clearly visible on the face X-ray). There are no other bone abnormalities or soft tissue detectable. A computed tomography (CT) scan of the right hip was prescribed as an adjunct and revealed an eccentric metaphyseal-diaphysealosteolytic lesion breaking the cortical without periosteal reaction (C).

**Figure 1:**
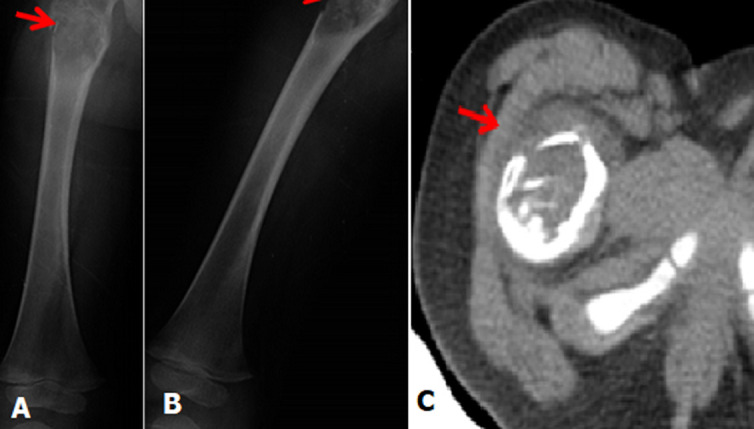
A) frontal femur X-ray; B) lateral femur X-ray; C) right hip CT scan: showing an eccentric osteolytic metaphyseal-diaphyseal lesion disrupting the cortex without periosteal reaction at the level of the right femur

